# Serum Neutralizing Activities from a Beijing Homosexual Male Cohort Infected with Different Subtypes of HIV-1 in China

**DOI:** 10.1371/journal.pone.0047548

**Published:** 2012-10-18

**Authors:** Mingshun Zhang, Yanmei Jiao, Shixia Wang, Lu Zhang, Zuhu Huang, Yuxin Chen, Hao Wu

**Affiliations:** 1 Jiangsu Province Key Laboratory in Infectious Diseases, The First Affiliated Hospital of Nanjing Medical University, Nanjing, China; 2 Department of Infectious Diseases, The First Affiliated Hospital of Nanjing Medical University, Nanjing, China; 3 China-US Vaccine Research Center, The First Affiliated Hospital of Nanjing Medical University, Nanjing, China; 4 Department of Microbiology and Immunology, Nanjing Medical University, Nanjing, China; 5 Department of Infectious Disease, Beijing Youan Hospital, Beijing, China; 6 Department of Medicine, University of Massachusetts Medical School, Worcester, Massachusetts, United States of America; University of Toronto, Canada

## Abstract

Protective antibodies play a critical role in an effective HIV vaccine; however, eliciting antibodies to block infection by viruses from diverse genetic subtypes remains a major challenge. As the world’s most populous country, China has been under the threat of at least three major subtypes of circulating HIV-1 viruses. Understanding the cross reactivity and specificities of serum antibody responses that mediate broad neutralization of the virus in HIV-1 infected Chinese patients will provide valuable information for the design of vaccines to prevent HIV-1 transmission in China. Sera from a cohort of homosexual men, who have been managed by a major HIV clinical center in Beijing, China, were analyzed for cross-sectional neutralizing activities against pseudotyped viruses expressing Env antigens of the major subtype viruses (AE, BC and B subtypes) circulating in China. Neutralizing activities in infected patients’ blood were most capable of neutralizing viruses in the homologous subtype; however, a subset of blood samples was able to achieve broad neutralizing activities across different subtypes. Such cross neutralizing activity took 1–2 years to develop and CD4 binding site antibodies were critical components in these blood samples. Our study confirmed the presence of broadly neutralizing sera in China’s HIV-1 patient population. Understanding the specificity and breadth of these neutralizing activities can guide efforts for the development of HIV vaccines against major HIV-1 viruses in China.

## Introduction

Infection with HIV-1 in humans leads to the production of serum antibodies against HIV’s envelope glycoprotein (Env). Several studies have systematically analyzed HIV-1 positive sera and discovered that not all HIV-1 infected patient sera achieve the same potency or breadth of neutralizing activities against primary HIV-1 viruses and only a fraction of infected sera can achieve broad neutralization against viruses from different genetic subtypes [1] [2] [3] [4] [5]. Specificities of such protective sera have been mapped to a limited number of epitopes that mediate broad and potent serum neutralization [6] [7]. These studies were conducted mainly with several subtypes of viruses dominating in the United States, United Kingdom, Thailand, and Africa and many of the subjects were infected via heterosexual transmission [1] [2] [3] [4] [5] [8].

However, HIV-1 isolates circulating throughout the world demonstrate great diversity. In China’s AIDS endemic, there are three major HIV-1 subtypes: Thai-B, CRF07/08_BC, and CRF01_AE [9], in addition to other less prevailing subtypes. Limited studies have described whether sera from people infected with one of these three subtypes can neutralize viral isolates from the other two subtypes. If such cross reactivity exists, the antibody specificities that may be responsible for broad neutralizing activities are unknown. Such information is valuable to HIV-1 vaccine development in countries such as China where multiple, equally important circulating viral subtypes have to be covered during vaccination.

In the current study, sera from a cohort of homosexual men were analyzed for neutralizing activities against pseudotyped viruses expressing Env antigens representing the major subtype viruses circulating in China. Key specificities responsible for the neutralizing activities have also been mapped in this exploratory study. Our study confirmed the presence of broadly neutralizing sera in this patient population and provided a scientific basis to develop HIV vaccines to achieve broad protection against major HIV-1 viruses in China. Such information is also useful for researchers to understand the global pattern of neutralizing antibodies (NAb) in HIV-1-infected patients, possibly leading to better control of the AIDS epidemic throughout the world.

## Methods

### HIV-1-infected Patients and Blood Sample Collection

Blood samples were collected from 36 homosexual male patients from the AIDS Clinic at Youan Hospital, Beijing, China in the summer of 2009. These patients had various lengths of confirmed diagnosis of HIV-1 infection.

### Ethics Statement

Written informed consent was obtained from all study participants. Sera and plasma samples were collected from the study subjects. They were diagnosed with HIV-1 infection but had not been on regular antiviral treatment prior to the start of the study. This study was carried out in strict accordance with the requirements for clinical studies established by the Capital Medical University, China. The protocol was approved by Youan Hospital’s Ethics Review Committee.

### Isolation of HIV-1 *env* Genes and *env* Sequence Genotyping

RNA was extracted from patient plasma samples using a QIAmp Viral RNA kit (Qiagen, CA). The envelope gene fragment covering C2∼V5 was amplified with the access RT-PCR system (Promega, #A1250). Specific primers (Table S1) used to perform the nested-PCR include outer sense primer TF1 (5′ -ATg ggA TCA AAg CCT AAA gCC ATg TgT-3′), outer anti-sense primer TR1 (5′ - gCg CCC ATA gTg CTT CCT gCT -3′), inner sense primer TF2 (5′ - ATT Agg CCA gTA gTA TCA ACT CAA -3′), and inner anti-sense primer TR2 (5′ - ATA TCT CCT CCT CCA ggT CTg AA -3′). The PCR products were sequenced by Invitrogen (Shanghai, China). The genotype of each envelope was determined using the PhyloPlace software from the US NIH HIV sequence database.

### Neutralization Assay

The neutralization assay was performed using a single round of infection in TZM-bl cells in 96-well plates, as previously described [10] [11]. Pseudoviruses were added at 200 TCID50/well and incubated with a mAb, HIV-1 patient serum, or a negative control normal human serum with proper dilutions at 37°C for 60 min. TZM-bl cells were then seeded at 10,000 cells/well in a final concentration of 20 µg/mL DEAE dextran. Plates were incubated at 37°C for 48 hours and developed with luciferase assay reagent per the manufacturer’s instructions (Promega). Neutralization was calculated as a percent reduction in luciferase activity in the presence of mAb or human sera compared to the luciferase activity induced by the virus in the absence of antibody {1− [RLU + mAb or immune sera]/[RLU – mAb or immune sera]}×100 [11] [12]. The reciprocal neutralizing antibody titers of human sera that achieved 50% reduction in luciferase activity (IC50) were also determined.

### Peptide Absorption Assay

To examine the potential contribution of V3-specific or membrane proximal external region (MPER)-specific antibodies to the neutralizing abilities of patient sera, V3 or MPER peptide absorption of HIV-1 patient sera was performed prior to neutralization, as described previously [11]. In this study, the subtype B consensus V3 peptide (CTRPNNNTRKSITHGPGRAFYTTGDIIGDIRQAHC) or Group M consensus MPER peptides were used. The MPER peptides include six peptides of 15-mer sequences with 11 amino acids overlap covering both 2F5 and 4E10 epitopes spanning the 35 amino acid MPER region (LIEESQNQQEKNEQELLALDKWASLWNWFDITNWL). One peptide (V3) or a mixture of six peptides (MPER) were added at a final concentration of 30 µg/ml to serially diluted serum samples and incubated at 37°C for 60 min. Then HIV-1 pseudovirus was added to the sera-peptide mixture for 37°C for 60 min. TZM-bl cells were then seeded at 10,000 cells/well in a final concentration of 20 µg/ml DEAE-dextran. Plates were incubated at 37°C for 48 hours before being developed with the luciferase assay reagent according to the manufacturer’s instructions (Promega). The percent reduction in neutralization was calculated as follows: [1 - (NAb titer in the presence of peptide)/(NAb titer in the absence of peptide)].

### Virus Capture Competition Assay

In order to analyze the antibody specificity of patient sera, HIV-1 viral capture competition assays were performed against known HIV-1 neutralizing monoclonal antibodies, including CD4bs-specific b12, VRC01 and VRC03 [7], and glycan-specific 2G12 and quaternary epitope-specific PG9 [13], as previously described [11]. Pseudotyped virions in this assay express both HIV-1 JR-FL Env and vesicular stomatitis virus (VSV) G protein. The VSV G protein mediates the entry of virions into the target cell line TZM-bl irrespective of any neutralizing activity against the HIV envelope present in the sera, thus providing a sensitive readout of captured virus as previously described [14]. The capture of pseudovirions by HIV-1 mAbs will be blocked if there is a competing antibody in the patient sera. Specifically, microwells were coated with individual mAbs (5 µg/ml) overnight and were then washed and blocked with 3% bovine serum albumin in PBS. Graded dilutions of patient sera were added to the virus, and the virus-serum mixtures were then added to mAb-coated ELISA wells for 3 hours, followed by washing with PBS. TZM-bl cells were overlaid, and 48 hours later, infection was measured by assaying luciferase activity. The reciprocal serum dilution that inhibited mAb-mediated virus capture by 50% was recorded.

### Statistical Analysis

Fisher’s exact test was used to determine the statistical differences in NAb among patient sera collected at various time points and their subtype specificities. Correlations between neutralization breadth and V3 peptide absorption or between neutralization breadth and antibody titers competing with neutralizing mAb IgG1 b12 were analyzed using Spearman’s rank correlation.

## Results

### Features of the HIV-1 Infected Cohort

Blood samples were collected from 36 male homosexual patients with a well-documented history of HIV-1 infection and most have the dates of their first diagnosis of HIV infection. Samples were collected during one of the patients’ regular clinic visits as a part of routine monitoring of their disease condition. The average age of this group was 30 years with an average blood CD4+ T cell count of 502 (Table S2). The period of time from diagnosis to sample collection ranged from 0.2–4.4 years, with 16 patients having less than a 1-year history of infection, 10 between 1–2 years’ history, and four with longer than 2 years’ history. Six patients could not provide reliable information on the exact length of infection.

**Figure 1:**
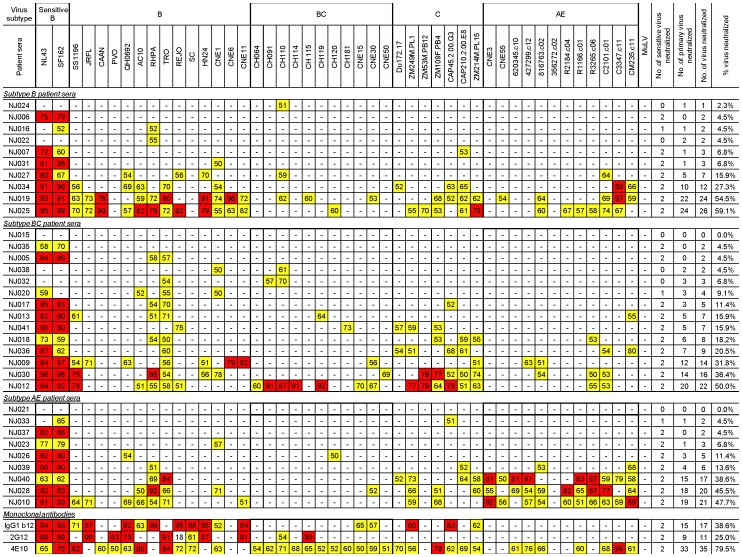
NAb activities against HIV-1 pseudotyped viruses. Neutralization was shown as the percent inhibition of virus infection at 1∶20 dilution of the patient sera or using 10 µg/ml of monoclonal antibodies (b12, 2G12, and 4E10). “−“ indicates that percent of neutralization was below 50%. Percent of neutralization between 50–75% or greater than 75% are highlighted in yellow or red, respectively. The HIV-1 pseudotyped viruses used for neutralization assays are shown in the second row from top from “NL4-3” to “CM235.c11” with subtype indicated above the virus panel. “MuLV” is the murine leukemia virus used as negative control for neutralization assays. “NJ0XX”s in the far left column indicates individual patient serum samples; patient HIV subtypes are indicated above each group of samples. Known neutralizing monoclonal antibodies, IgG1 b12, 2G12 and 4E10, served as positive controls for neutralization assays.

### Identification of Viral Subtypes in Infected Plasma

The C2-V5 region of the HIV-1 *env* gene was amplified from patient plasma by reverse transcription (RT) and nested-polymerase chain reaction (PCR), using primers described in Table S1, and the gene products were sequenced. These *env* gene sequences were analyzed for HIV-1 subtypes based on the NIH HIV sequence database. The majority of *env* gene sequences isolated from this group of patients belonged to subtypes B/ThaiB, BC, and AE, which are the three major circulating HIV-1 subtypes in China (Fig. S1 and Table S3). Subtypes for *env* gene products from two patients in this cohort were unidentifiable and, therefore, were not included for NAb analysis.

**Figure 2:**
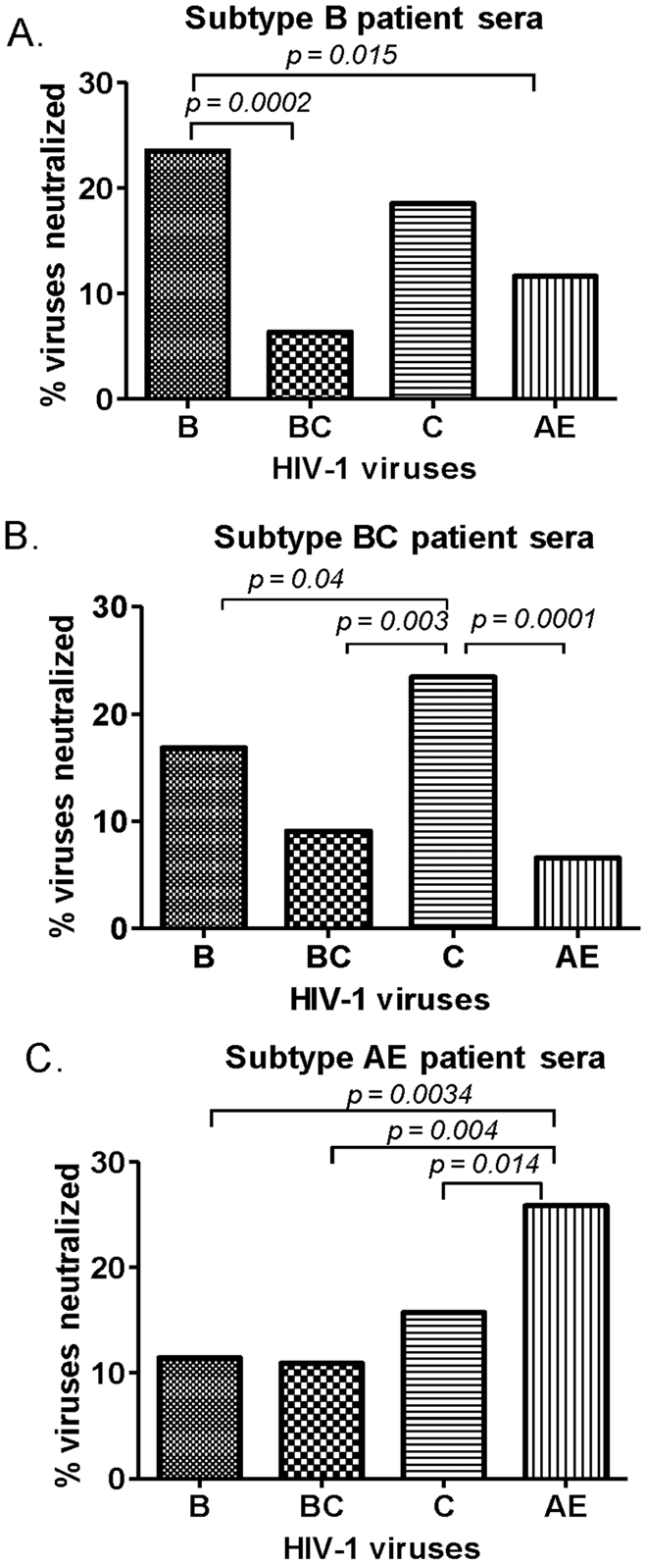
Subtype specificity of NAb responses identified in HIV-1 infected patients’ sera. Neutralization against pseudotyped viruses expressing primary Env from subtypes B, BC, C, and AE isolates were tested. Percentage of neutralization by patients’ sera (at 1∶20 dilution) infected with one of three viral subtypes (B, BC, and AE as shown in panels A, B, and C, respectively) were calculated. The significant differences (p<0.05) of neutralizing activities between different groups of patient sera are indicated.

### Neutralizing Activities in Infected Patients’ Sera

NAb analysis was conducted against pseudotyped virus infection in TZM-bl cells (Fig. 1). A panel of pseudotyped viruses expressing 44 primary HIV-1 Env antigens was included in this study: 14 from subtype B viruses, 11 from subtype BC, 7 from subtype C, and 12 from subtype AE. Two pseudotyped viruses expressing either an Env antigen from a T-cell line adapted (TCLA) virus (NL4-3) or an Env from a sensitive primary virus (SF162) were included as controls. The breadth and potency of such activities varied widely among this cohort. Given the large differences in NAb titers, a summary of neutralizing activities was compiled by using the same serum dilution at 1∶20. The percent inhibition at this serum dilution was reported to indicate the relative strength of such activities when inhibition less than 50% was considered negative in the current assay (Fig. 1).

**Figure 3:**
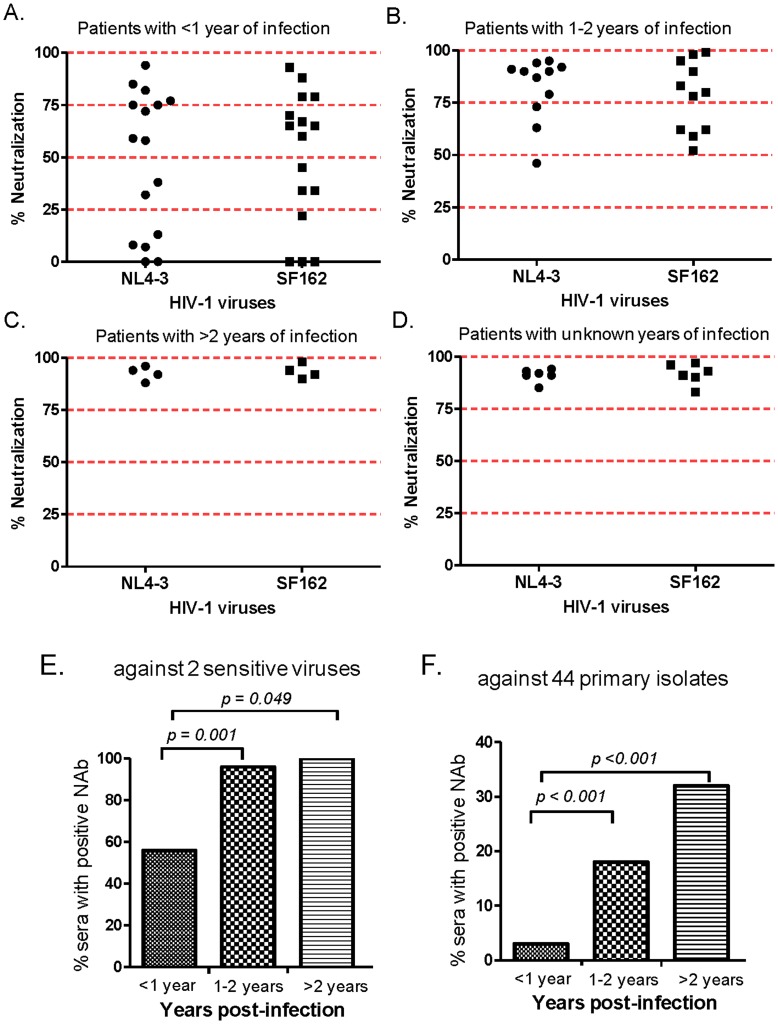
Improvement of NAb responses after one year of HIV-1 infection. Panels A, B, C and D represent the percent (%) of neutralization at 1∶20 serum dilution against two sensitive subtype B viruses (SF162 and NL4-3), within 1 year, between 1–2 years, longer than 2 years of infection, or time of initial infection unknown. Panels E and F represent the percentage of HIV-1 infected patient sera that showed positive NAb responses (using IC50 at 1∶20 serum dilution as the cut-off) based on length of infection (<1 year, 1–2 years, and >2 years). Panel A shows neutralizing activities against two sensitive viruses (SF162 and NL4-3) and panel B against 44 pseudotyped viruses expressing primary Env antigens (see Fig. 1). The levels of statistical difference between patient sera with different lengths of infection are indicated, which were determined by Fisher exact test.

Among 34 serum samples where *env* gene subtypes were mapped, 25 showed NAb responses against both sensitive viruses (SF162 and NL4-3), three (NJ016, NJ020, and NJ033) could only neutralize one virus, and the remaining six samples (NJ024, NJ022, NJ015, NJ038, NJ032, and NJ021) could not neutralize either virus. Among these nine samples that could not neutralize either sensitive virus, all, except one (NJ016), had a history of infection for less than 1-year, indicating that sera from patients with a shorter time period after infection were unable to generate effective neutralizing activities. In support of this view, the only two serum samples (NJ015 and NJ021) that could not neutralize any viruses expressing primary Env in our analysis were among these nine samples. The remaining seven samples had neutralizing activities against no more than 1–3 primary Env pseudotyped viruses.

**Figure 4:**
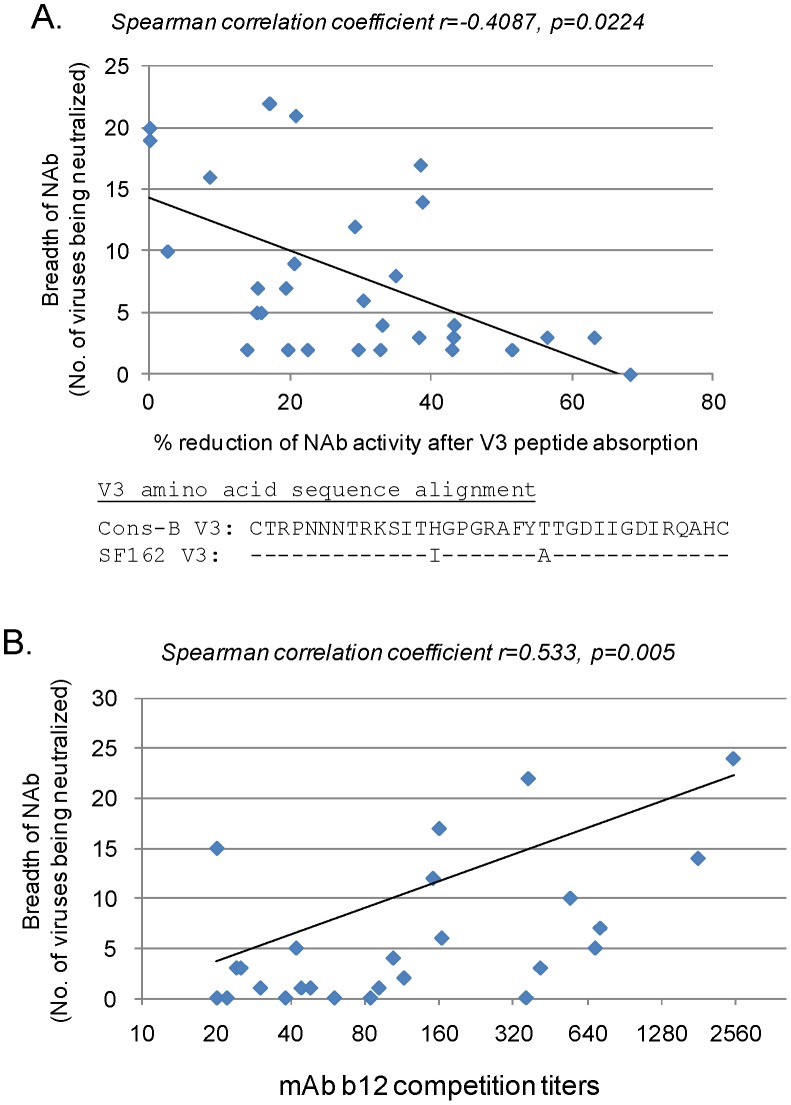
Analysis of possible correlations between neutralization breadth in HIV-1 patient sera and selected known neutralizing epitopes V3 and CD4bs. A. Analysis on the relationship between the reduction of neutralizing activities after V3 peptide absorption in patient sera and the breadth of neutralization with the same patient sera. Percent reduction of neutralizing activities against pseudotyped SF162 was tested with individual patient serum (1∶20 dilution) after absorption with the subtype B consensus V3 peptide. The alignment of subtype B consensus (Cons-B) and SF162 V3 sequence is shown. The number of primary Env pseudotyped viruses (total 44) neutralized by each individual patient serum were calculated as shown in Fig. 1. Correlations and significance were determined using the Spearman method. B. The relationship between antibody titers to compete against neutralizing mAb b12 and the breadth of neutralization (number of viruses being neutralized) in the same patient sera was analyzed. Correlations and significance were determined using the Spearman method.

For the 25 patient sera samples that were able to neutralize both of the sensitive viruses, only two (NJ006 and NJ035), both from patients with less than 1-year infection, could not neutralize any other primary Env pseudotyped viruses, suggesting that once NAb are elicited, sera can neutralize not just the sensitive viruses, but also additional primary Env viruses.

**Figure 5:**
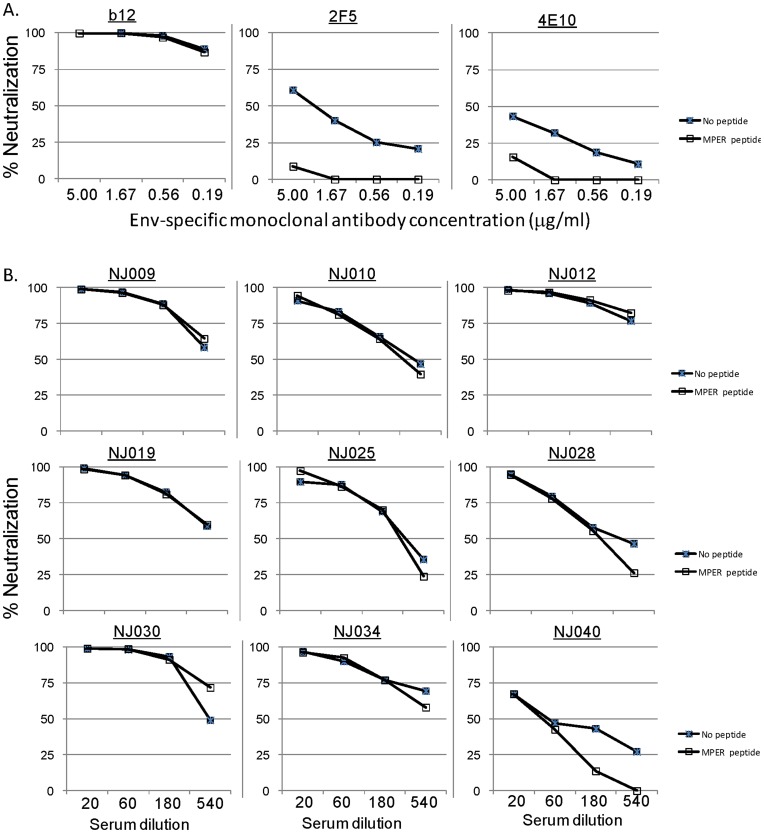
MPER peptide inhibition of neutralizing activities of Env-specific mAbs and HIV-1 patient sera. A: MPER inhibition of NAb activities of mAbs (b12 against CD4bs, 2F5 and 4E10 against MPER) as control; B: MPER inhibition of NAb activities of patient sera with relative broad NAb activities (NJ009, NJ010, NJ012, NJ019, NJ025, NJ028, NJ030, NJ034, and NJ040).

As reported in the past, these serum samples showed preferred neutralization against viruses from the autologous subtype (Fig. 1). By using the high percentage inhibition (75–100%) as a more reliable measurement on the strength of neutralizing activities (red boxes in Fig. 1), there were nine “hits” in subtype B sera against subtype B Env pseudotyped viruses, none against subtype BC, one hit against subtype C, and two hits against subtype AE Env. Sera from subtype BC had a combined nine “hits” against subtypes BC and C, three against subtype B, and none against subtype AE Env. Finally, sera from patients infected with subtype AE viruses, had a strikingly high level of “hits” (N = 10) against the subtype AE Env, but only two against subtype B, and none against subtypes C and BC Env.

**Table 1:** CD4bs mAb competition antibody titers.

Patient serum	No. of positive NAb against primary viruses	gp120 CD4bs specific mAb			
		b12		VRC01	VRC03
*Narrow NAb* *					
NJ015	0	–	–		–
NJ035	0	–	–		–
NJ024	1	44	–		–
NJ033	1	–	–		–
*Broad NAb* *					
NJ040	15	20	–		29
NJ004	17	160	–		–
NJ019	22	368	20		116
NJ025	24	2502	156		152

“−“ indicates that the antibody titer is below detection level at 1∶20 serum dilution.

* significant difference for CD4bs mAb competition antibodies between patient sera with Narrow or Broad NAb activities (p = 0.0014 by Fisher exact test).

When a more quantitative analysis was conducted by combining all positive neutralizing activities together (both red and yellow (50–74% inhibition) boxes, serum samples from subtype B patients had the highest percentage of neutralization against subtype B viruses, which was significantly higher than the subtypes BC (p = 0.002) and AE (p = 0.015) (Fig. 2A). Subtype BC patient sera neutralized more subtype C than the subtypes B, BC, or AE (p = 0.04, p = 0.003, and p = 0.0001, respectively) (Fig. 2B). Serum samples from subtype AE patients neutralized more subtype AE viruses than subtypes B, C, and BC (p = 0.0034, p = 0.004, and p = 0.014 respectively) (Fig. 2C). Our data demonstrated that, in general, HIV-1-infected patient sera tend to have the best neutralizing activities against viruses from the autogolous subtype but there is a low frequency of cross-subtype neutralizing activities, more likely with sera from patients infected with subtypes B and BC viruses.

**Table 2:** Antibody titers competing against Env-specific mAbs 2G12 and PG9 in patient sera with relative broad NAb activities.*

Env-specific mAbs	Subject ID								
	NJ009	NJ010	NJ012	NJ019	NJ025	NJ028	NJ030	NJ034	NJ040
2G12	–	–	–	35	–	–	–	–	–
PG9	–	–	–	27	–	–	–	–	–

* The mAb 2G12 competition assays were conducted against SF162 while the PG9 competition assays were performed against NL4-3.

“−“ indicates the competition antibody titers were below the detection level (1∶20).

A subset of patient sera showed quite broad neutralizing activities (Fig. 1). Among 10 patients infected with subtype B viruses, four (40%) could neutralize viruses from 3–4 subtypes. Among 14 patients infected with subtype BC viruses, seven sera (50%) were able to neutralize viruses from 3–4 subtypes. Among 10 patients infected with subtype AE viruses, four (40%) were able to neutralize 3–4 subtypes. However, even among these cross-subtype neutralizing serum samples, only five samples (NJ019, NJ025, NJ030, NJ012, NJ028), representing 15% of total serum samples tested, could neutralize more than 50% of this 44-virus panel across all four subtypes.

### Delayed Emergence of Broadly NAb Responses

Since many serum samples included in the current study did not show broad NAb responses until later in infection, it was interesting to examine whether a longer period of infection in the host may be needed in order to elicit broad NAb responses. Using subtype B virus-infected patient sera as an example, Fig. 3 (A–D) shows that NAb titers increased after one year’s infection against two sensitive viruses (SF162 and NL4-3); similar results were found using sera that showed positive neutralizing activities. When all serum samples were included, sera with positive neutralizing activities against the two sensitive viruses increased from less than 50% of the total samples for those with <1 year’s infection to close to 100% for those with >2 years’ infection (Fig. 3E). The same pattern was observed against 44 primary Env pseudotyped viruses (Fig. 3F), most of which were more resistant viruses. On average, less than 5% of the neutralizing events against the primary Env viruses were observed with sera from patients infected <1 year, but this rate increased to close to 20% with 1–2 years’ infection, and to more than 30% after two years’ infection (Fig. 3F).

### Neutralizing Antibody Specificity Analysis

In order to further understand the specificity of anti-Env antibody responses that may be responsible for the breadth of NAb against primary HIV-1 viruses in this cohort of HIV-1-infected patients, two types of assays were conducted. In the first assay, serum samples were treated with HIV-1 subtype B consensus V3 peptide before the same neutralization study was conducted against SF162, which can be neutralized by majority of the patient sera in this study. Decreases in NAb responses upon the treatment of this V3 peptide were observed although a significant number of sera only had a moderate drop (20–40%) with V3 peptide treatment (Fig. 4A). An overall inverse-correlation was observed between the reduction of neutralizing activity upon treatment with V3 peptide and the breadth of NAb responses (r = −0.409, p = 0.022). These results suggested that patient sera with broader neutralizing activities were less V3-dependent while those with narrower neutralizing activities were more V3-dependent against SF162 tested in this study.

Nine serum samples with relative broad NAb activities were further treated with HIV-1 Group M consensus MPER peptides (six peptide pool spanning both 2F5 and 4E10 epitopes) before the same neutralization study was conducted against SF162. Decreases in NAb activities upon treatment of MPER peptides were only observed in patient NJ040 and not in the other eight sera samples (NJ009, NJ010, NJ012, NJ019, NJ025, NJ025, NJ028, NJ030, and NJ034) (Fig. 5B). To control the MPER absorption assays, CD4bs-specific mAb b12 and MPER-specific mAbs 2F5 and 4E10 were also treated with MPER peptides before neutralization. As expected, MPER peptides could inhibit the NAb activities of MPER-specific mAbs 2F5 and 4E10 but could not inhibit the NAb activity of gp120-specific mAb b12 (Fig. 5A). These results suggest that patient sera with broader neutralizing activities are not MPER-dependent.

The next study examined the antibody components in patients’ sera that can compete against b12, a well-known CD4 binding site (CD4bs) neutralizing monoclonal antibody. Here, a significant correlation was observed between titers of b12-competing antibody responses and the number of primary viruses being neutralized (p = 0.005, r = 0.533) (Fig. 4B).

To further support this finding, four broadly neutralizing sera from the current patient cohort were analyzed for the presence of antibodies that may compete against two newly discovered broadly neutralizing human mAbs, VRC01 and VRC03, which also target the CD4bs (Table 1). Two sera infected with subtype B viruses, NJ019 and NJ025, showed broad neutralizing activities against HIV-1 viruses from different subtypes (Table 1). Both showed positive competing antibodies against VRC01 and VRC03 although their relative titers were much lower than the b12-competing antibodies (Table 1). NJ040, a serum infected with subtype AE virus, had only low level VRC03-competing antibodies, and the fourth serum, NJ004, infected with subtype C, did not have detectable levels of antibodies competing against either VRC01 or VRC03. Interestingly, both NJ040 and NJ004 sera had low levels of b12-competing antibody responses. In contrast, four serum samples (one from subtype B, two from subtype C, and one from subtype AE), which had very poor neutralization against the primary viruses and did not have significant b12-competing antibodies, also failed to show the presence of competing antibodies against both VRC01 and VRC03. Although this analysis was conducted on only a few samples, the results demonstrate the presence of VRC01- and VRC03-competing antibodies in broadly neutralizing sera and, more likely, in sera rich in b12-competing antibodies.

To further examine the specificity of patient sera with relative broad NAb activities, we conducted mAb competition assays against glycan-specific mAb 2G12 and quaternary epitope-specific mAb PG9 (Table 2). Among nine patient sera with relative broad NAb, only patient NJ019 had detectable low titers of antibodies competing against mAbs 2G12 and/or PG9. This implied that 2G12-like or PG9-like antibodies were not in significant amounts in this set of patient sera when compared to CD4bs-mAb-like antibodies.

Using the same virus capture competition assay, two mAbs recognizing glycosylated Env antigens and quarternary structure (2G12 and PG9) were tested to compete against HIV-infected sera (Table 2). Except for one serum, which showed low titers against both mAbs, most of other broadly neutralizing antibodies could not compete against these two mAbs.

## Discussion

In this study, we reported neutralizing activities in a group of HIV-1-infected homosexual male patients from Beijing, China. Like many other countries, China has not escaped the AIDS endemic that has occurred over the past two decades. While initially HIV-1 infections have been mainly observed in injection drug users in certain border provinces, the latest data suggest an increase in HIV-1 infections in urban areas through sexual transmission [9] [15], including an alarming rate of homosexual transmission [16] [17] [18] [19]. Following these new epidemiological trends and gaining a better understanding of immune responses among people infected with HIV-1 in China will provide useful information on the dynamics and ultimate control of the AIDS epidemic in China. At the same time, we need to be mindful on the recent RV144 trial data [20], which showed that NAb response was not one of the immune correlates for protection. More vaccination studies are needed to fully define any protective roles that NAb may play for HIV-1 vaccines.

It is well-documented in the literature that the genetic makeup of HIV-1 in China is quite complex due to the way various viral subtypes infect different risk groups within the population. Three main subtypes have dominated HIV-1 infections in China including CRF07/08_BC (BC), subtype B’ (B’), and CRF01_AE (AE). Previous studies have suggested that each of these subtypes was associated with a particular mode of transmission [21] [22]: intravenous drug use with BC viruses, commercial blood and plasma donation with B’ viruses, and sexual transmission with AE viruses. Furthermore, it was also suggested that AE viruses were found more in those infected via homosexual transmission [21]. However, in our current study, we have identified all three major subtypes in the homosexual male population indicating different subtypes of viruses have reached major urban areas, including Beijing. Given the relatively small size of this cohort, the exact rates of infection by each of the three major subtypes were not determined.

Co-circulation of multiple subtypes of HIV-1 is a challenge to vaccine development in China. Furthermore, China offers a unique geographic model to examine cross-neutralizing antibody activities against well-defined subtypes in the same region. While early studies did not reach a consensus on the presence of subtype-specific humoral responses in HIV-1-infected people [23] [24] [25] [26], recent reports on the analysis of patient serum antibodies in China support the preferred neutralization of viruses of the autologous subtype [27] [28] [29]. Data in the current report further confirm that HIV-1-infected patient sera are, indeed, more capable of neutralizing viruses in the same subtype compared to viruses from other subtypes. Even for those sera with cross-subtype neutralizing activities, they exhibited either a higher titer or a higher frequency when it came to neutralizing viruses from the autologous subtype.

However, our data also suggest that such subtype specificity is not absolute. Many sera showed various degrees of cross-neutralizing activities against viruses from other subtypes. Between 40–50% of the tested sera were able to neutralize viruses in more than three subtypes but the real broadly neutralizing sera, which could neutralize more than 50% of the virus panel, represented only 15% of the sera; results from this study also showed that it will take more than 1–2 years’ time to develop such breadth. Previous studies on HIV-infected patients in China either did not pay particular attention to identifying broadly neutralizing sera [27] [28] [29] or only used pooled sera to determine those that greatly reduced the sensitivity to detect such high quality neutralizing sera [17]. The finding of such naturally occurring broad neutralizing sera made it feasible to elicit similar broad NAb through vaccination.

It is particularly critical to understand the specificity of antibody responses responsible for the induction of broadly NAb against viruses in all three subtypes. Our data suggest that there are antibodies against more than one neutralizing epitope present in these sera and CD4bs antibodies may play a critical role in the breadth of neutralizing activities. Our results did not identify antibodies similar to either 2G12 or PG9. Peptide absorption using a MPER peptide was not able to reduce the NAb identified in our HIV patient sera. However, the current study is only exploratory and detailed studies are needed to examine neutralizing activity against pseudotyped viruses with selected mutations on those existing Env to further map out critical sites involved in neutralizing viruses in both autologous and heterologous subtypes; such information will guide the design of Env vaccine formulations to elicit broadly neutralizing activities.

Our data also point to a major challenge in eliciting NAb responses, i.e. the lengthy period required to detect such antibody responses in naturally-infected hosts. We realize the limitation of the cross-sectional nature of our data as we did not follow these patients over time with multiple sampling time points. It is not clear whether such a long process is needed to let the viruses evolve in order to allow for the development antibodies against a more diverse viral population, or whether a long B-cell maturation process is needed to produce high-quality antibody responses against the same key epitopes. In the former situation, future vaccines will need to be based on “polyvalent’ principles to expand the breadth of immunogens. In the latter scenario, more effective immunization approaches may be needed, such as the use of adjuvants to selectively accelerate the necessary steps for the production of high quality antibodies. Both approaches can be tested in future vaccine studies to determine if similar cross-subtype antibody responses can be reproduced.

## Supporting Information

Figure S1 **Phylogenetic tree analysis of the C2–C4 region of HIV-1 Env genes included in the current study.** Each sequence included in the analysis is labeled. Red-colored branches show representative reference HIV-1 *env* gene sequences from GenBank. HIV-1 subtype clusters are marked. A neighbor joining tree model was used for the analysis.
(DOC)

Table S1 **Primers for RT-PCR.**
(DOC)

Table S2 **Cohort of HIV-1 infected patents.**
(DOC)

Table S3 **Env gene subtyping.**
(DOC)
