# Correction: Serum Neutralizing Activities from a Beijing Homosexual Male Cohort Infected with Different Subtypes of HIV-1 in China

**DOI:** 10.1371/annotation/288be20c-88d0-450e-98d2-22599de22f81

**Published:** 2012-12-28

**Authors:** Mingshun Zhang, Yanmei Jiao, Shixia Wang, Lu Zhang, Zuhu Huang, Yuxin Chen, Hao Wu, Shan Lu

In the online version of this article, author Dr. Shan Lu was not included in the author byline. He should be listed as the eighth author and affiliated with: **1** Jiangsu Province Key Laboratory in Infectious Diseases, The First Affiliated Hospital of Nanjing Medical University, Nanjing, China, **3** China-US Vaccine Research Center, The First Affiliated Hospital of Nanjing Medical University, Nanjing, China, **6** Department of Medicine, University of Massachusetts Medical School, Worcester, Massachusetts, United States of America. He should also be noted as the corresponding author, whose email address is: shan.lu@umassmed.edu

This information is present in the PDF version of the article.

