# Supplementary material for: Serum Neutralizing Activities from a Beijing Homosexual Male Cohort Infected with Different Subtypes of HIV-1 in China
Source: PLoS One. 2012 Oct 18;7(10):e47548. doi: 10.1371/journal.pone.0047548 (PMC3475692; doi:10.1371/journal.pone.0047548)
Supplement: Table S1 — Primers for RT-PCR. (DOC) [file pone.0047548.s002.doc]

Table S1. Primers for RT-PCR

| **Primer** | **Sequence** | **Position in HXB2** |
| --- | --- | --- |
| *Primers targeting env gene* | |  |
| TF1 | ATg ggA TCA AAg CCT AAA gCC ATg TgT | 6557 - 6583 |
| TR1 | gCg CCC ATA gTg CTT CCT gCT | 7819 - 7799 (*reverse*) |
| TF2 | ATT Agg CCA gTA gTA TCA ACT CAA | 6975 - 6998 |
| TR2 | ATA TCT CCT CCT CCA ggT CTg AA | 7648 - 7626 (*reverse*) |
| *Primers targeting Gag gene* | |  |
| GAG-F2 | ATg ggC gCg AgA gCg TCA RTA TTA A | 790 - 814 |
| R1399 | CCA CAT TTC CAA CAg CCC TTT TT | 2039 - 2017 (*reverse*) |
| U208 | ggg AAA AAA TTCA ggT TAA ggC | 836 - 856 |
| R1196 | CCC TgA CAT gCT gTC ATC ATT TCT TCT | 1844 - 1818 (*reverse*) |
