# Supplementary material for: Serum Neutralizing Activities from a Beijing Homosexual Male Cohort Infected with Different Subtypes of HIV-1 in China
Source: PLoS One. 2012 Oct 18;7(10):e47548. doi: 10.1371/journal.pone.0047548 (PMC3475692; doi:10.1371/journal.pone.0047548)
Supplement: Table S2 — Cohort of HIV-1 infected patents. (DOC) [file pone.0047548.s003.doc]

Table S2. Cohort of HIV-1 infected patents

| **Patient ID** | **Age** | **Year of infection** | **CD4 counts** |
| --- | --- | --- | --- |
| NJ022 | 37 | 0.2 | 632 |
| NJ015 | 39 | 0.2 | 478 |
| NJ021 | 22 | 0.2 | 686 |
| NJ038 | 26 | 0.2 | 256 |
| NJ035 | 23 | 0.3 | 305 |
| NJ032 | 35 | 0.3 | 751 |
| NJ023 | 27 | 0.4 | 411 |
| NJ024 | 29 | 0.4 | 569 |
| NJ033 | 49 | 0.4 | 410 |
| NJ020 | 24 | 0.5 | 595 |
| NJ002 | 37 | 0.6 | 474 |
| NJ014 | 24 | 0.6 | 689 |
| NJ007 | 23 | 0.7 | 605 |
| NJ027 | 32 | 0.7 | 340 |
| NJ037 | 20 | 0.7 | 336 |
| NJ006 | 53 | 0.8 | 804 |
| NJ028 | 36 | 1.1 | 200 |
| NJ039 | 28 | 1.1 | 409 |
| NJ041 | 23 | 1.1 | 511 |
| NJ016 | 25 | 1.2 | 649 |
| NJ018 | 23 | 1.2 | 238 |
| NJ040 | 42 | 1.2 | 242 |
| NJ036 | 26 | 1.3 | 622 |
| NJ025 | 53 | 1.4 | 317 |
| NJ005 | 28 | 1.6 | 643 |
| NJ031 | 34 | 1.9 | 734 |
| NJ030 | 26 | 2.0 | 859 |
| NJ004 | 39 | 2.3 | 222 |
| NJ013 | 24 | 2.4 | 537 |
| NJ012 | 27 | 4.4 | 924 |
| NJ009 | 28 | Unknown | 263 |
| NJ010 | 27 | Unknown | 283 |
| NJ017 | 27 | Unknown | 229 |
| NJ019 | 22 | Unknown | 18 |
| NJ026 | 29 | Unknown | 261 |
| NJ034 | 28 | Unknown | 101 |
| Average | 29.8 | 1.04 | 502.47 |
