# Supplementary material for: Serum Neutralizing Activities from a Beijing Homosexual Male Cohort Infected with Different Subtypes of HIV-1 in China
Source: PLoS One. 2012 Oct 18;7(10):e47548. doi: 10.1371/journal.pone.0047548 (PMC3475692; doi:10.1371/journal.pone.0047548)
Supplement: Table S3 — Env gene subtyping. (DOC) [file pone.0047548.s004.doc]

Table S3. Env gene subtyping

| **Patient ID** | **Env subtype** |
| --- | --- |
| NJ014 | AE, CRF01 |
| NJ023 | AE, CRF01 |
| NJ026 | AE, CRF01 |
| NJ029 | AE, CRF01 |
| NJ033 | AE, CRF01 |
| NJ037 | AE, CRF01 |
| NJ039 | AE, CRF01 |
| NJ040 | AE, CRF01 |
| NJ002 | B, CRF15-01B |
| NJ006 | B |
| NJ010 | B, CRF15-01B |
| NJ016 | B, CRF15-01B |
| NJ019 | B, CRF15-01B |
| NJ021 | B |
| NJ022 | B |
| NJ024 | B |
| NJ025 | B, CRF15-01B |
| NJ027 | B, CRF15-01B |
| NJ028 | B |
| NJ031 | B |
| NJ034 | B |
| NJ004 | BC, CRF07 |
| NJ005 | BC, CRF07 |
| NJ007 | BC, CRF08 |
| NJ008 | BC, CRF08 |
| NJ009 | BC, CRF08 |
| NJ012 | BC, CRF07 |
| NJ013 | BC, CRF08 |
| NJ017 | BC, CRF07 |
| NJ018 | BC, CRF08 |
| NJ020 | BC, CRF08 |
| NJ030 | BC, CRF08 |
| NJ032 | BC, CRF07 |
| NJ035 | BC, CRF07 |
| NJ036 | BC, CRF07 |
| NJ038 | BC, CRF08 |
| NJ041 | BC, CRF07 |
