# Supplementary material for: Serum Neutralizing Activities from a Beijing Homosexual Male Cohort Infected with Different Subtypes of HIV-1 in China
Source: PLoS One. 2012 Oct 18;7(10):e47548. doi: 10.1371/journal.pone.0047548 (PMC3475692; doi:10.1371/journal.pone.0047548)
Supplement: Supplementary file 4 [file pone.0047548.s001.doc]

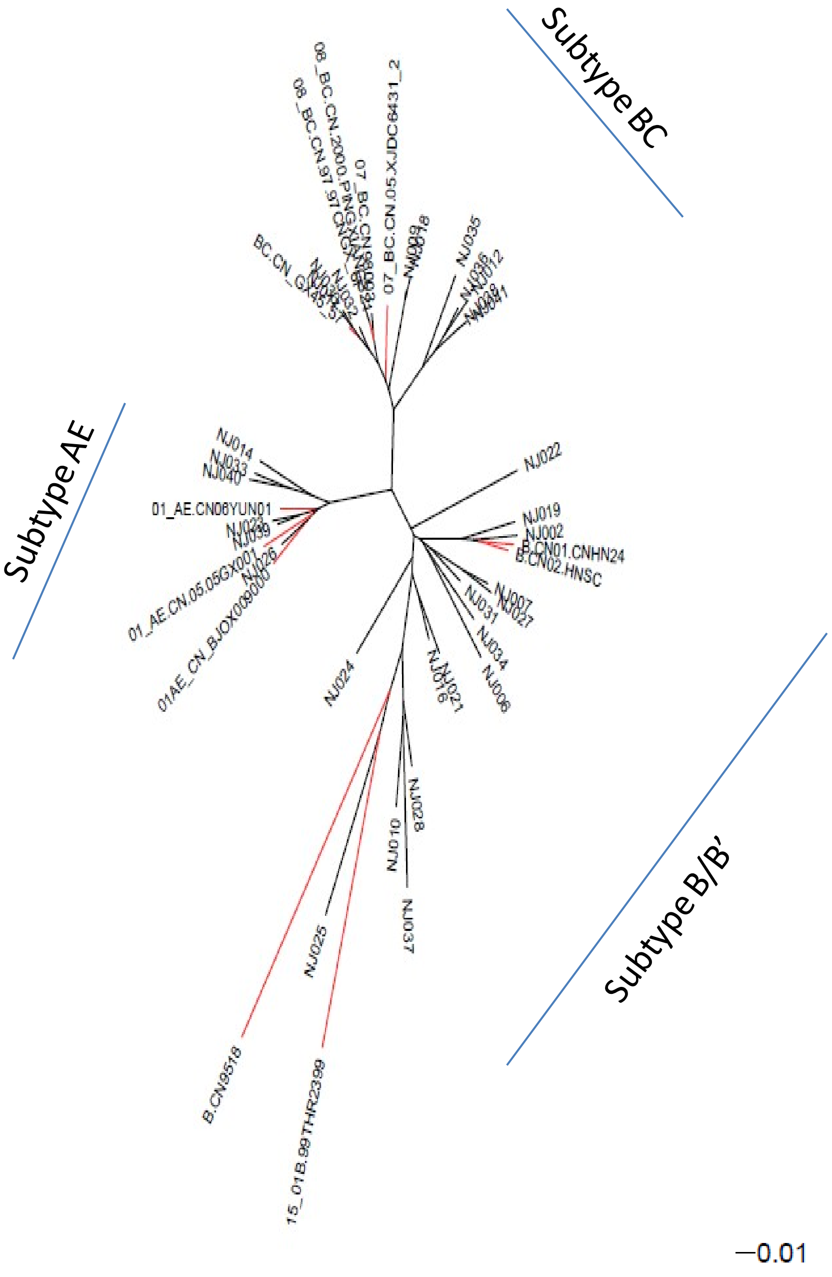
Subtype BC
Subtype AE
Subtype B/B’
07_BC.CN.05.XJDC6431_2
NJ035
NJ022
NJ014
NJ033
NJ040
01_AE.CN06YUN01
01_AE.CN.05.05GX001
01AE_CN_BJOX009000
NJ019
NJ002
B.CN01.CNHN24
B.CN02.HNSC
NJ007
NJ027
NJ031
NJ034
NJ006
NJ024
NJ021
NJ016
NJ028
NJ010
NJ037
NJ025
B.CN9518
15_01B.99THR2399
0.01
